# Supplementary material for: Evolution of fungal and non-fungal eukaryotic communities in response to thermophilic co-composting of various nitrogen-rich green feedstocks
Source: PLoS One. 2023 May 31;18(5):e0286320. doi: 10.1371/journal.pone.0286320 (PMC10231794; doi:10.1371/journal.pone.0286320)
Supplement: Attachment — Submitted filename: Response_to_Reviewers.docx [file pone.0286320.s001.docx]

The Editorial Board

PlosOne

10^th^ April, 2023

Dear Editor,

**RE: REVIEWERS’ COMMENTS ON MANUSCRIPT PONE-D-22-30019] - [EMID:8685918cb01db8f4] AND HOW THEY HAVE BEEN ADDRESSED**

I would like to appreciate the opportunity to submit a revised version of the manuscript entitled “**Evolution of fungal and non-fungal eukaryotic communities in response to thermophilic co-composting of various nitrogen-rich green feedstocks**” to be considered for publication in your Journal as an original article. Kindly also note that the referred line numbers are on the “Revised Manuscript with Track Changes” file.

We highly appreciate and welcome your comments and those of the reviewers and have addressed the points raised during the review process as follows;

**Reviewer Comments:**

**Reviewer #1:**

Thank you very much for the authors efforts to respond to my comments, but in the still you should improve your manuscript, there are some further comments.

**Reviewer comment No. 1:** line 75: Please correct "in invitro" by dropping "in".

**Authors’ response:**

The word ‘in’ has been dropped and replaced with ‘under’.

**Reviewer comment No. 2:** Line 113,145: write "icipe" in capital letters.

**Authors’ response:**

This is acknowledged and changed throughout the manuscript.

**Reviewer comment No. 3:** Table 2: Authors should indicate the meaning of each symbol and the unit: for example, Carbon (C), ditto for all others (N, P .....).

**Authors’ response:**

This has been corrected as advised.

**Reviewer comment No. 4:** line 261: regarding the fungus class nomenclature, the authors should remove the italic character style. please review the overall manuscript.

**Authors’ response:**

## This has been changed throughout the text as advised.

**Reviewer comment No. 5:** You still need to demonstrate the novelty of your manuscript, it is not clear.

**Authors’ response:**

The authors aver that the novelty of the study is on lines 25-29, 72-80. The rationale for this study is articulated within the manuscript in lines 93-101, where the justification for studying the diverse green materials has been presented “These materials have, however, been reported to have antimicrobial activity in in-vitro experiments. Furthermore, the phytochemical analysis has shown differences in their complexities, thus possibly requiring various periods to break down. Therefore, it is necessary to understand these materials' influence on the biological and physical-chemical stability of compost” and lines 65-75 “Some of these materials have been reported under invitro studies as having inhibitory properties on fungal and non-fungal organisms [13, 14]. The phytochemical complexities of Lantana camara and Tithonia diversifolia have been previously studied. Lantana camara has been reported to contain more complex polymers and thus possibly requires more microbial categories to break down into agriculturally useful material. For example, the phytochemical composition analysis of L. camara showed 23.3% crude fiber, 26% cellulose, 16.2% lignin, and 21% hemicellulose, while T. diversifolia contains 11.2%, 17%, 7%, and 16% of these elements respectively [15, 16, 17]. Furthermore, the different complexities of the composting material could require assorted composting times to produce a biogeochemically stable product. It is, therefore, necessary to evaluate the influence of composting time on the biological and physical-chemical quality of compost”

The importance of Eukaryotes in the ecosystems and their limited study is enumerated in lines 51-56 “Though less studied, eukaryotes are an important microbial category in the ecosystem with diverse feeding and genetic guilds [6, 7]. Eukaryotes degrade recalcitrant material such as Carbon-rich polymers, thus contributing to nutrient cycling in the compost environment [8]. The complex structure and function of a large number of compost microorganisms are directly affected by various environmental factors such as temperature, moisture, Carbon/Nitrogen ratio, and pH, among others”.

The use of next-gen sequencing to study eukaryotes is justified in lines 124-130 “Numerous studies have assessed the microbial prokaryotic community in the composting process using culture-dependent and culture-independent methods [2, 4, 6, 10]. However, there is still a limited understanding of eukaryotic communities’ structure in the composting process, concerning the composition and complexity of the composting materials especially utilizing high-throughput sequencing technology. The effect of the Lantana camara and Tithonia diversifolia on microbial community structure in complex ecosystems such as the compost environment has not also been done, despite their widespread adoption by farmers as soil nutrient amendment materials”.

Moreover, lines 29-30, and 57-59 have been added to increase clarity.

**Reviewer 2**

Reviewer #2: (No Response)

**Authors’ response:**

The authors appreciate the great input from the reviewer.

We reiterate our appreciation to the reviewers for their kind comments which have helped improve the manuscript and hope that we have adequately responded to all the comments raised regarding this manuscript in readiness for its publication in your journal.

Thank you for your consideration.

Yours Sincerely

Felix Matheri

Department of Biochemistry, Microbiology, and Biotechnology, Kenyatta University,

P.O Box 30772-00100 Nairobi.
